# Liver Impairment and Hematological Changes in Patients with Chronic Hepatitis C and COVID-19: A Retrospective Study after One Year of Pandemic

**DOI:** 10.3390/medicina57060597

**Published:** 2021-06-10

**Authors:** Bianca Cerbu, Stelian Pantea, Felix Bratosin, Iulia Vidican, Mirela Turaiche, Stefan Frent, Ema Borsi, Iosif Marincu

**Affiliations:** 1 Department of Infectious Diseases, Victor Babes University of Medicine and Pharmacy, 300041 Timisoara, Romania; ionitabiancaelena@yahoo.com (B.C.); felix.bratosin7@gmail.com (F.B.); iulia.georgianabogdan@gmail.com (I.V.); mirela.turaiche@gmail.com (M.T.); imarincu@umft.ro (I.M.); 2 Surgical Clinic 2 Department, Victor Babes University of Medicine and Pharmacy, 300041 Timisoara, Romania; 3 Center for Research and Innovation in Precision Medicine of Respiratory Diseases, Department of Pulmonology, “Victor Babes” University of Medicine and Pharmacy, 300041 Timisoara, Romania; frentz.stefan@umft.ro; 4 Department of Internal Medicine—Hematology, Victor Babes University of Medicine and Pharmacy, 300041 Timisoara, Romania; ema.budai@yahoo.com

**Keywords:** COVID-19, SARS-CoV-2 infection, hepatitis C virus, liver impairment, mortality risk

## Abstract

*Background and Objectives*: The COVID-19 pandemic is an ongoing public health emergency. Patients with chronic diseases are at greater risk for complications and poor outcomes. The objective of this study was to investigate the liver function abnormalities and clinical outcomes in patients with COVID-19 and chronic hepatitis C. *Materials and Methods*: This retrospective, single-center study was conducted on a cohort of 126 patients with a history of hepatitis C, confirmed with COVID-19 between 01 April 2020 and 30 December 2020. Several clinical outcomes were compared between patients with active and non-active HCV infection, and the risks of liver impairment and all-cause mortality in active HCV patients were analyzed using a multivariate logistic regression model. *Results*: Among 1057 patients under follow-up for chronic HCV infection, 126 (11.9%) were confirmed with COVID-19; of these, 95 (75.4%) were under treatment or achieved SVR, while in the other 31 (24.6%), we found active HCV replication. There was a significantly higher proportion of severe COVID-19 cases in the active HCV group as compared to the non-active HCV group (32.2 vs. 7.3%, *p* < 0.001). Multivariate analysis showed that age, sex, alanine aminotransferase, C-reactive protein, procalcitonin, and HCV viral load were significant independent risk factors for liver impairment and all-cause mortality. The length of stay in hospital and intensive care unit for COVID-19 was significantly higher in patients with active HCV infection (*p*-value < 0.001), and a higher proportion of these patients required mechanical ventilation. *Conclusions*: Active HCV infection is an independent risk factor for all-cause mortality in COVID-19 patients.

## 1. Introduction

Severe acute respiratory syndrome coronavirus 2 (SARS-CoV-2) emerged in late December 2019, with the epicenter in Wuhan, China, and has since infected more than 135 million people, causing over 2.9 million deaths worldwide [1]. The COVID-19 pandemic has proven to be a serious challenge for the Romanian healthcare system, with more than 1 million infections and approximately 25,000 deaths being reported in the country at the time of writing. Comorbidities are observed in 20–30% of COVID-19 patients, while the proportion increases to 50–80% in patients with severe COVID-19 [2]. Since the beginning of the pandemic, the effect of comorbidities has been widely discussed; however, the impact of coronavirus infection on the liver has not been well studied. Furthermore, several medications used for the treatment of COVID-19 may cause hepatotoxicity [3].

In 2018, there was a reported incidence of 37,527 cases of chronic hepatitis C in the EU/EEA Member States [4], with a total prevalence of 19 million cases [5] and 71 million cases worldwide [6]. As the global prevalence of hepatitis C virus (HCV) and SARS-CoV-2 infections are geographically variable, epidemiological data across Europe may help improve our understanding of the reciprocal impact of SARS-CoV-2 and HCV. Simultaneously, we have investigated the prevalence of COVID-19 among HCV patients who achieved cure using a sofosbuvir/velpatasvir (SOF/VEL) combination antiviral treatment in all cases. The interplay between a pre-existing liver disease and SARS-CoV-2 infection may be important for patients’ outcomes, since chronic hepatitis C is still a health burden in many European countries. Even though the exact impact of SARS-CoV-2 infection on the liver is unknown, abnormalities in liver biochemistry are typical in COVID-19 cases, arising in 15–65% of patients infected with SARS-CoV-2 [7,8].

In the present study, we focused on the assessment of hematological changes and liver function abnormalities, as evidenced by routine laboratory testing in patients co-infected with HCV and SARS-CoV-2, and the relationship between COVID-19 severity and progression of liver disease. We describe the possible relationship between the clinical course, laboratory findings, and outcome of 126 patients admitted in our department with documented HCV infection and COVID-19 disease.

## 2. Materials and Methods

We conducted a retrospective cohort study to evaluate the risk for liver impairment and all-cause mortality in COVID-19 patients with active HCV infection. We collected clinical and laboratory data using the archived records of consecutive patients with COVID-19 at the Infectious Diseases and Pulmonology Hospital, “Victor Babes” Timisoara from 1 April 2020 to 1 September 2020. Among the patients confirmed with SARS-CoV-2 infection within this time frame, we identified those cases with a documented history of HCV infection. The group of patients with HCV and SARS-CoV-2 co-infection was further divided into active and non-active HCV infection groups according to their history of hepatitis C diagnosis and treatment. Patients with liver cirrhosis were excluded from the study. An active HCV infection was defined by an HCV RNA viral load greater than 0.015 (U/L × 10^3^). Subsequently, various clinical parameters and outcomes, such as length of hospital stay, number of intensive care unit (ICU) admissions, length of ICU stay(s), as well as routine blood parameters, including white blood cell count (WBC), red blood cell count (RBC), hemoglobin levels (Hb), platelet count (PLT), liver function tests such as alanine transaminase (ALT), aspartate transaminase (AST), alkaline phosphatase (ALP), albumin and total plasma proteins, total bilirubin, gamma-glutamyltransferase (GGT), L-lactate dehydrogenase (LDH), and prothrombin time (PT); HCV viral load, procalcitonin, C-reactive protein (CRP), and all-cause mortality were assessed and compared between those with and without active HCV infection. Serial monitoring of the laboratory profile was performed according to the clinical progress of each individual patient. Liver impairment was defined as the alteration above normal ranges for the liver function tests: ALT, AST, ALP, GGT, LDH, PT, albumin, total plasma proteins, total bilirubin.

The patients enrolled were at least 18 years old, the intensive care unit (ICU) admissions were required for a rapid progression of severe pneumonia with pulmonary infiltrates covering over 50% of the lung fields, severe dyspnea, oxygen saturation ≤ 93% despite face mask oxygen supplementation, or paCO2 > 55 mmHg in patients without chronic obstructive pulmonary disease (COPD). SARS-CoV-2 infection was confirmed by the detection of viral RNA in nasopharyngeal secretion using specific reverse-transcription polymerase chain reaction (RT-PCR) test. COVID-19 infection was further classified into three clinical forms: mild, moderate, and severe. The mild form of COVID-19 infection was defined as a low-grade fever without pneumonia. The moderate form was defined by the presence of fever and signs of non-severe pneumonia, without need for oxygen treatment. The criteria used to define the severe cases included respiratory failure requiring mechanical ventilation, a respiratory rate greater than 30/min, oxygen saturation of hemoglobin measured by pulse-oximetry (SpO_2_) < 90%, coagulation disorders, failure of other organs requiring admission to the ICU, and ground-glass opacities involving more than 50% of the lungs on the chest X-ray or CT scan. The treatment scheme for all patients admitted to our clinic with COVID-19 infection included a standard association of remdesivir, dexamethasone, azithromycin antibiotic prophylaxis, and anticoagulation using enoxaparin, since studies confirmed the correlation between COVI-19 and thrombosis [9], with adjustments being made based on patient profile.

The Local Committee of Ethics for Scientific Research of “Dr. Victor Babes” Infectious Diseases and Pulmonology Clinical Hospital Timisoara operates under art provisions 167 of Law no. 95/2006, art. 28, chapter VIII of order 904/2006 and with EU GCP Directives 2005/28/EC, International Conference on Harmonisation of Technical Requirements for Registration of Pharmaceuticals for Human Use (ICH), and with the Declaration of Helsinki—Recommendations Guiding Medical Doctors in Biomedical Research Involving Human Subjects. The current study protocol received the ethical approval on the 10th of September 2020, with the approval number 1292.

We compared clinical and laboratory data of COVID-19 patients with and without active HCV infection using the IBM SPSS v.26 statistical software. The χ^2^ test and Fisher’s exact test were used for categorical variables and Student’s *t*-test or Mann–Whitney *U*-test for continuous variables. The independent risk factors associated with the COVID-19-related clinical outcomes of liver impairment and mortality were identified using a logistic regression model. Risk factors are reported as odds ratios (ORs) with 95% confidence intervals (CIs). The significance threshold was set for α = 0.05.

## 3. Results

**Figure 1:**
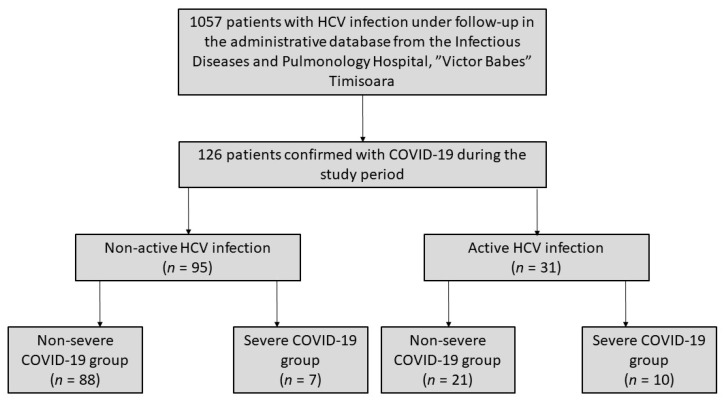
Flowchart of the study cohort. Of 1057 patients with HCV infection, 126 were confirmed with COVID-19 during the study period. A total of 95 patients were categorized into the non-active HCV infection, while the other 31 patients were having an active HCV infection. Among the non-active HCV infection 88 and 7 patients were classified into the non-severe and severe COVID-19 infection groups, respectively, while from the active HCV infection 21 and 10 patients were classified into the non-severe and severe COVID-19 infection groups, respectively.

Among 1057 patients infected with HCV that were under follow-up in our clinic, 126 (11.9%) were confirmed with COVID-19 between 1 April 2020 and 1 September 2020. Of these, 95 patients (75.4%) were under treatment with the SOF/VEL scheme or achieved sustained virologic response (SVR), while in the other 31 (24.6%), we found active HCV replication. In the group of patients with a non-active HCV infection, 88 (92.6%) had a mild or moderate form of COVID-19 infection, while seven patients (7.4%) presented a severe form of disease. In the group of patients with active HCV infection, 21 (67.7%) had a mild or moderate form of COVID-19, while 10 (32.3%) presented with severe COVID-19 infection (Figure 1).

When comparing the proportion of severe COVID-19 cases in the active and non-active HCV infection groups, we found a statistically significant higher prevalence in the active HCV group (32.2 vs. 7.3%, χ^2^ = 12.40, *p*-value = 0.0004).

The baseline demographics of the study population are shown in Table 1. The majority of patients in the active HCV group were male (58.1 vs. 35.7%, *p*-value < 0.0001) and belonged to the 41–60 years age group (51.6 vs. 30.6%, *p*-value < 0.0001). There was no difference between groups in the prevalence of reported comorbidities. The patients in the active HCV group experienced more signs and symptoms, fatigue being significantly more prevalent in the active HCV group (83.8 vs. 64.2%, *p*-value < 0.0001), followed by myalgia (54.8 vs. 34.7%, *p*-value < 0.0001), and fever (87.0 vs. 72.6%, *p*-value < 0.0001). Oxygen saturation levels on admission were most commonly above 95% in the non-active HCV infection group (83.1 vs. 45.1% *p*-value < 0.0001), while in the active HCV group, we found significantly higher proportions of patients with oxygen saturation levels between 90–95% and below 90% (19.3 vs. 7.3%, *p*-value < 0.0001 and 35.4 vs. 9.4%, *p*-value < 0.0001, respectively). A lung involvement consisting in > 40% of the lung parenchyma affected by the SARS-CoV-2 infection on chest computed tomography (CT) was significantly more prevalent in patients with active HCV infection (32.2 vs. 7.3%, *p*-value < 0.0001).

Table 2 compares the clinical outcomes between non-active and active HCV infection groups. The rate of hospitalization and length of stay were significantly higher in the active HCV group (61.2 vs. 40.0%, *p*-value < 0.0001 and 28 vs. 21 days, *p*-value = 0.0142, respectively). Among the patients hospitalized for COVID-19, 10 patients (52.6%) in the active HCV group and seven patients (18.4%) in the non-active HCV group were admitted to the ICU (*p*-value < 0.0001). The proportion of patients requiring mechanical ventilation (26.3 vs. 13.1%, *p*-value = 0.0007) and all-cause mortality were higher in the active HCV group (48.3 vs. 11.5%, *p*-value < 0.0001).

A multivariate analysis (Table 3) was conducted to assess the risk factors for liver impairment and all-cause mortality in COVID-19 patients with chronic hepatitis C. Age over 60 years (OR = 2.51, 95%CI 1.43–3.02), male gender (OR = 2.36, 95%CI 1.41–3.15), ALT (OR = 3.17, 95%CI 1.61–3.98), procalcitonin (OR = 2.88, 95%CI 1.45–2.95) levels, and HCV viral load were all independent risk factors for liver impairment. Furthermore, an age over 60 years (OR = 8.27, 95%CI 5.14–13.5), male gender (OR = 1.66, 95%CI 1.27–2.20), ALT level (OR = 1.45, 95%CI 1.18–2.19), and HCV viral load (OR = 2.46, 95%CI 1.17–3.56) were also independent risk factors for all-cause mortality.

Significant changes in blood parameters (Table 4.) were observed in both study groups, with mean WBC (*p* < 0.0001), ALT (*p* < 0.0001), AST (*p* < 0.0001), ALP (*p* = 0.012), LDH (*p* < 0.0001), PT (*p* < 0.0001), procalcitonin (*p* < 0.0001), CRP (*p* < 0.0001), and HCV viral load (*p* < 0.0001) being significantly higher and platelet count significantly lower (*p* < 0.0001) in COVID-19 patients with active HCV infection.

## 4. Discussion

Previous studies [10] investigated the changes in liver function in patients with COVID-19, assessing the routine blood parameters in liver function tests. Partially their observations were consistent with our findings, showing significant increases in ALT, AST, LDH, CRP, and coagulation tests in severe COVID-19 cases and a decrease in albumin, when compared to mild cases of infection. However, these findings did not differ when compared to hospitalized patients with community-acquired pneumonia. Moreover, no liver parameter demonstrated any significant relationship as a risk factor with severe COVID-19 cases and mortality, since only old age and the neutrophil–lymphocyte ratio (NLR) were identified as independent risk factors. On the other hand, our study focused only on patients with chronic HCV infection, thus, the higher likelihood of worsened liver function compared to the general population. Other larger studies [11] demonstrated that impaired liver function is a prognostic factor for poor outcomes in patients with COVID-19, consequently increasing the length of hospital stay, similar to our observations. The disease severity in patients with chronic liver dysfunction was closely associated with older age, male gender, and high body mass index (BMI). The authors assumed that the liver injury may have been actually induced by hepatotoxic effect of drugs used to treat the SARS-CoV-2 infection.

Another study analyzed the possible pathologic mechanisms standing behind impaired liver function in patients with COVID-19 [12]. However, there were no conclusive arguments and data to clarify whether these changes may have been a result of the direct action of SARS-CoV-2 on the liver. It was reported that patients with chronic liver disease are more likely to die by acute-on-chronic liver failure and respiratory failure, while suffering from COVID-19. In the same direction, Grove J. et al. [13] studied the molecular mechanisms involved in the coronaviruses hepatotropism, which was earlier hypothesized as hepatic injury and inflammation being able to potentiate SARS-CoV-2 hepatotropism by modulating viral receptor expression, since the angiotensin-converting enzyme 2 (ACE2) receptor was earlier identified as an interferon-inducible gene in human respiratory epithelia [14]. Additionally, the SARS-CoV receptor-binding domain (RBD) binds to the ACE2 receptors allowing the virus to enter targeted cells [15], while also inhibiting the ACE2 enzyme, which typically protects the lungs from damage [16]. Since ACE2 receptors are found in biliary and hepatic epithelial cells, the liver appears to be vulnerable to SARS-CoV-2 infection [17]. The study described how the high-density lipoprotein scavenger receptor B type 1 (SR-B1) helps facilitate ACE2-dependent coronavirus attachment in vitro, reminiscent of HCV infection.

The possibility of drug-induced hepatotoxicity was also taken into consideration in our research. Other studies explored this hypothesis [18], showing that patients with HCV infection were more likely to experience drug-induced liver damage, particularly when receiving highly active antiretroviral therapy [19]. Thus, all hospitalized patients in our cohort received a treatment scheme consisting of lopinavir/ritonavir and darunavir, based on the existing national guidelines. Inconsistently, 103 (81.7%) patients also received antibiotic therapy comprising of azithromycin, moxifloxacin, vancomycin, meropenem, ceftriaxone, or levofloxacin, according to the alleged bacterial pathogens involved, therapies that may have potential hepatotoxic consequences [20].

Our study is limited by the retrospective cohort study design, since there were multiple healthcare workers engaged in patient care; therefore, measuring risk factors and effects through the database is likely to be less reliable and accurate when compared to a prospective cohort design where these factors can be carefully controlled.

Finally, the current study brings novel findings and conclusions regarding patients with active and non-active chronic HCV infection without liver cirrhosis, who are subsequently infected with the SARS-CoV-2 virus, since this topic has not been fully studied to this date. Various studies [21,22] observed higher mortality rates in COVID-19 patients with liver cirrhosis caused by chronic hepatitis B and C, although a recent meta-analysis [23] concluded that chronic liver disease, including chronic HCV infection, appears to have a small impact on the COVID-19 progression to a severe form of disease, findings that are inconsistent with our observations.

## 5. Conclusions

In conclusion, active HCV infection was associated with more severe disease and higher mortality in patients co-infected with SARS-CoV-2, HCV viral load being an independent risk factor for all-cause mortality and liver impairment. The severity of liver impairment was associated with poor clinical outcomes in COVID-19 patients. Further research is required to confirm our findings in prospective studies and larger samples. Our results suggest that close monitoring and careful treatment for active-HCV patients with COVID-19 are needed to avoid health deterioration and fatal outcome.

## Figures and Tables

**Table 1:** Patient characteristics.

Characteristics	Non-Active HCV (*n* = 95)	Active HCV (*n* = 31)	*p*-Value
Age (years)			
18–40	16 (16.8)	3 (9.7)	<0.0001
41–60	29 (30.6)	16 (51.6)	<0.0001
>60	50 (52.6)	12 (38.7)	<0.0001
Sex, male	34 (35.7)	18 (58.1)	<0.0001
Comorbidities			
High blood pressure	51 (53.7)	17 (54.8)	0.8474
Heart failure	12 (12.6)	5 (16.1)	0.1733
Ischemic heart disease	16 (16.8)	7 (22.5)	0.1428
Diabetes	21 (22.1)	5 (16.1)	0.0914
Stroke	6 (6.3)	2 (6.4)	0.4517
Cancer	8 (8.4)	4 (12.9)	0.2691
Renal failure	8 (8.4)	5 (16.1)	0.0679
Signs and Symptoms			
Anosmia	42 (44.2)	14 (45.1)	0.8074
Ageusia	37 (38.9)	12 (38.7)	0.7721
Fatigue	61 (64.2)	26 (83.8)	<0.0001
Cough	67 (70.5)	25 (80.6)	0.0501
Myalgia	33 (34.7)	17 (54.8)	<0.0001
Diarrhea	19 (20.0)	11 (35.4)	0.0468
Headache	48 (50.5)	20 (64.5)	0.0042
Fever	69 (72.6)	27 (87.0)	<0.0001
SaO2 on admission			
>95%	79 (83.1)	14 (45.1)	<0.0001
90–95%	7 (7.3)	6 (19.3)	<0.0001
<90%	9 (9.4)	11 (35.4)	<0.0001
Lung Involvement on Chest CT			
<20%	71 (74.7)	12 (38.7)	<0.0001
20–40%	17 (17.8)	9 (29.0)	0.0013
>40%	7 (7.3)	10 (32.2)	<0.0001

HCV—hepatitis C virus; SaO2—oxygen saturation level.

**Table 2:** Clinical outcomes.

Variables	Non-Active HCV	Active HCV	*p*-Value
Hospital admission			
Number of patients	38 (40.0)	19 (61.2)	<0.0001
Length of stay (days)	21.3 ± 13.1	28.1 ± 17.4	0.0142
ICU admission			
Number of patients	7 (18.4)	10 (52.6)	<0.0001
Length of stay (days)	14.4 ± 10.2	9.5 ± 8.7	<0.0001
Mechanical ventilation	5 (13.1)	5 (26.3)	0.0007
ECMO	1 (2.6)	0 (0.0)	0.0648
All-cause mortality	11 (11.5)	15 (48.3)	<0.0001

HCV—hepatitis C virus; ECMO—extracorporeal membrane oxygenation.

**Table 3:** Multivariate analysis.

Variable	Liver Impairment		All-Cause Mortality	
	Adjusted OR	95% CI	Adjusted OR	95% CI
Age > 60 years old	2.51	1.43–3.02	8.27	5.14–13.5
Gender, male	2.36	1.42–3.15	1.66	1.27–2.20
ALT	3.17	1.61–3.98	1.45	1.18–2.19
Procalcitonin	2.88	1.45–2.95	1.02	0.88–2.13
HCV viral load	8.72	5.28–11.3	2.46	1.17–3.56

HCV—hepatitis C virus.

**Table 4:** Blood parameters.

Blood Parameters—Mean (SD)	Non-Active HCV (*n* = 95)	Active HCV (*n* = 31)	*p*-Value
WBC (x/mm^3^)	11,434 (4662)	14,890 (4285)	<0.0001
RBC (x/mm^3^)	4,455,509 (519,104)	4,574,418 (688,314)	0.9100
Hb (g/dL)	13.2 (1.5)	13.3 (1.7)	0.4298
PLT (x/mm^3^)	214,541 (76,778)	183,327 (71,225)	<0.0001
ALT (U/L)	34.1 (44.8)	121.4 (89.7)	<0.0001
AST (U/L)	22.8 (15.6)	104.5 (72.7)	<0.0001
ALP (U/L)	41.4 (12.8)	66.2 (13.3)	<0.0012
Albumin (g/dL)	4.5 (0.4)	4.1 (0.4)	0.4752
Total proteins (g/dL)	7.2 (2.0)	6.9 (1.8)	0.3821
Total bilirubin (g/dL)	1.1 (0.3)	1.2 (0.4)	0.0520
GGT (U/L)	12.2 (3.1)	13.7 (3.3)	0.0921
LDH (U/L)	255 (62.6)	320.5 (97.2)	<0.0001
PT (seconds)	11.2 (0.9)	14.7 (2.4)	<0.0001
Procalcitonin (ug/L)	0.2 (0.0)	0.5 (0.1)	<0.0001
CRP (mg/L)	10.5 (1.2)	74.9 (15.6)	<0.0001
HCV viral load (U/L × 10^3^)	0.011 (0.006–0.019)	103,650 (43,921)	<0.0001

## Data Availability

Data available on request.
